# Texas State Medical Association—Section on Practice

**Published:** 1891-03

**Authors:** 


**Texas State Medical Association, Section on Practice.**

—The Secretary of the Section on Practice of Medicine, Dr. B. F. Church, of Terrell, requests the JOURNAL to announce the following papers which have been contributed to the Section on Practice and will be read at the meeting at Waco: "*Pemphigus*" by Dr. O. Eastland, Wichita Falls; "How Remedies Affect the Human Economy When Applied to the Skin," by Dr. J. O. Lowrie, Coleman, Texas.

---

**Dr. W. L. York**, of Decatur has been appointed a member of the Judicial Council, vice Dr. Terhune, deceased, who had been appointed vice Dr. B. Saunders, resigned.

---

**The Ebbett House** Proprietors, Washington City, write the secretary of the T. S. M. A. that "special rates of $3 to $4 and up" will be made to Texas delegates to the American Medical Association which meets in that city May, 5th.

